# Evaluation of Clinical Meaningfulness of Fortasyn Connect in Terms of “Time Saved”

**DOI:** 10.14283/jpad.2024.55

**Published:** 2024-03-13

**Authors:** Samuel P. Dickson, A. Solomon, M. Kivipelto, T. Hartmann, A. M. J. van Hees, A. Brownlee, B. Haaland, C. H. Mallinckrodt, S. B. Hendrix

**Affiliations:** 1 Pentara Corporation, 2261 East 3300 South, Millcreek, UT 84109 USA; 2 Department of Neurology, Institute of Clinical Medicine, University of Eastern Finland, Kuopio, Finland; 3 Department of Clinical Geriatrics, Department of Neurobiology, Care Sciences and Society, Karolinska Institute, Huddinge, Sweden; 4 Theme Inflammation and Aging, Karolinska University Hospital, Huddinge, Sweden; 5 Ageing Epidemiology (AGE) Research Unit, School of Public Health, Imperial College London, London, UK; 6 Institute of Public Health and Clinical Nutrition, University of Eastern Finland, Kuopio, Finland; 7 Deutsches Institut für Demenz Prävention (DIDP), Medical Faculty, Saarland University, Homburg, Germany; 8 Department of Experimental Neurology, Saarland University, Homburg, Germany; 9 Danone Nutricia Research, Utrecht, The Netherlands

**Keywords:** Time saved, Alzheimer’s disease and mild cognitive impairment, Fortasyn Connect, Souvenaid, clinical relevance and meaningfulness, nutritional supplements

## Abstract

Assessment of meaningfulness in randomized clinical trials (RCTs) in Alzheimer’s disease (AD) is challenging, particularly in early disease. Converting clinical outcomes to disease progression time allows assessment of treatment effects using a metric that is understandable and meaningful: time. We demonstrate time savings assessments using meta time component tests (TCTs) in the LipiDiDiet multinutrient RCT. Dietary patterns are important for dementia prevention, likely due to individual cumulative nutrient effects. LipiDiDiet used a multinutrient (Fortasyn Connect) formulation in patients with prodromal AD, benefitting cognition (5-item composite NTB, effect 0.089), cognition and function (CDR-SB, −0.605), and slowing hippocampal atrophy (0.122 cm^3^). Meaningfulness of point differences is unclear. However, a combination TCT showed 9-month disease time savings at 24 months (38% slowing of disease time): 9.0, 10.5, and 7.2 months for NTB, CDR-SB, and hippocampal volume, underscoring the value of TCTs in AD RCTs and the need for continued validation of this approach.

## Introduction

The evaluation of the clinical relevance of outcomes in randomized clinical trials (RCTs) targeted towards Alzheimer’s disease (AD) represents a significant challenge (1–4). Recently, it has been concluded that treatments designed to slow the progression of the disease may require a reevaluation of anticipated therapeutic outcomes, and a more thorough analysis of the temporal dimension is necessary (3, 5). However, this proposition was solely based on hypothetical concepts and synthetic datasets. To address this issue, the present study employs meta time component tests (TCTs) to analyze actual clinical trial data as a real-world example of the implementation of this strategy.

Disease modifying AD therapies and interventions are expected to change disease progression trajectory by slowing the rate of clinical decline, but this poses analytic challenges. It may be optimal to commence treatment early in the disease continuum to optimize clinical benefit, but disease progression at early stages of disease is slow in most patients. Therefore, effect sizes will be small, even for highly effective therapies. This is mitigated by using a large sample size and / or long-term treatment and long follow-up periods in clinical trials. However, exposing many patients to long periods of placebo control, and the large cost of such trials, is problematic, especially for early phase trials.

**Figure 1:**
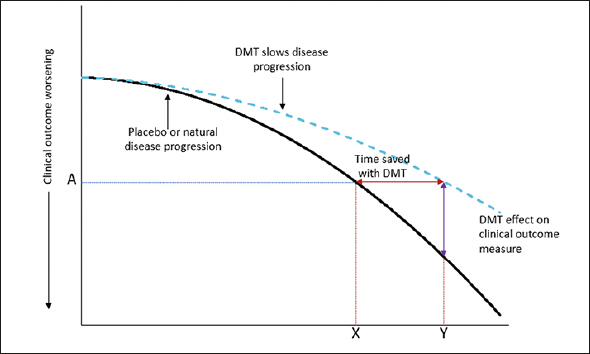
An illustration of a disease-modifying effect, whereby treatment delays disease progression Footnote: At time point Y, the change on the clinical outcome over time is less with active treatment than placebo (or natural disease progression). At this time point, the decline on the clinical outcome with active treatment (A) was reached at time point X with placebo. The difference between X and Y is the time gained with active treatment. As an example, a 25% reduction in the progression on an outcome measure with a DMT is equivalent to delaying disease progress by 3 months over 12 months of treatment. Adapted from Dickson, et al. 2023, Journal of Prevention of Alzheimer’s Disease – https://creativecommons.org/licenses/by/4.0/.

Treatment effects in the clinical trial environment are evaluated using a validated clinical scale to determine the difference between intervention and control groups in mean point change from baseline to endpoint. Graphically, this is assessment of the vertical difference between treatment groups (Fig 1, purple line). Whether this represents an overall percentage slowing in clinical decline with the intervention or the absolute point difference between intervention and control, it is not always intuitive to translate this vertical separation into a clinical effect of a treatment. Furthermore, this assessment creates challenges in comparing findings across studies that employed different outcome measures.

It has been proposed that translating the mean point change difference into a time component may have benefit in understanding the clinical relevance of an intervention effect (3, 5). This change shifts focus from absolute difference on a clinical outcome scale to the difference between intervention and control in the time to reach a specified degree of worsening, i.e., time saved with treatment. In other words, time saved is the horizontal difference between treatment groups (Fig 1, red line).

This intuitively understood “time saved” approach could provide a more easily and consistently interpreted measure because it directly assesses the time delay in patient decline in cognition, functional abilities, or global assessment. This is essential because this “time saved” due to active treatment allows patients to remain independent longer and participate more fully in daily life. That is, time saved is what is important to patients, families, care givers, and physicians.

Assessing time saved also provides time as a common measurement scale across differing endpoints. Different outcomes measure different disease pathology aspects, but when converted to time saved, the common measurement scale facilitates comparison of findings across outcomes, both within and between trials.

Fortasyn Connect is a multi-nutrient formulation containing docosahexaenoic acid, eicosapentaenoic acid, uridine monophosphate, choline, B-vitamins (B12, B6, folic acid), vitamin C, vitamin E, phospholipids, and selenium. Preclinical investigations showed that this specific formulation is neuroprotective and reduced AD-linked brain pathologies (6–13). Clinical trials have suggested that Fortasyn Connect’s clinical benefits are predominantly observed when the treatment is initiated early in the disease continuum, i.e., prodromal AD (14). Notably, the LipiDiDiet RCT reported significant differences in memory, cognitive and functional performance, and hippocampal atrophy in the active group compared to the placebo control group in a population of patients with prodromal AD according to IWG1 criteria (15, 16).

The current investigation endeavors to implement TCT to determine how cognitive, functional, global, and structural outcomes may translate into time saved. With the extended treatment duration, the existence of significant differences between the active and control arms, and the validation of biomarkers in the prodromal AD study population, the LipiDiDiet RCT provides a suitable dataset for the study’s objectives.

## Methods

### TCT Methodology

Time component tests (TCT) are applicable for assessing treatment effect only when the control group exhibits decline and when the treatment is expected to be disease modifying. The TCT used here relies on the least squares mean estimates from a primary analysis such as a mixed model for repeated measures (MMRM) over time rather than individual participant data (17–20). The mean change for each outcome at the end of the study is aligned horizontally with the mean change in the placebo group at an earlier time point, interpolating between visits if necessary. The distance between points where the mean changes correspond, the horizontal distance, is the estimated time savings. The same horizontal mapping approach is used to convert the mean plus one standard error and the mean minus one standard error to the time scale. The standard error of the time estimates is approximated as half of the difference between these upper and lower times. For detailed methods and statistical properties of TCT and associated standard error constructions, see Dickson et al. (21).

The TCT approach can be applied to multiple outcome measures, with results combined across outcomes on the time scale in a global statistical testing framework that accounts for the correlation between the outcomes included in the TCT, leading to a single statistical test across outcomes, a global TCT or gTCT. Here, we adopt an optimized approach to gTCT construction, which targets a minimum variance combination incorporating differences in uncertainty and interrelationships between endpoints based on quadratic programming (22). We minimize ω′Σω subject to the weights w being non-negative and summing to 1, where Σ is an estimate of the variance-covariance matrix of the treatment effects across endpoints. This optimized approach offers notable efficiency gains, particularly when sampling variability differs across endpoints. Detailed methods and statistical properties of gTCT constructions, including standard errors, can also be found in Dickson et al. (21).

### LipiDiDiet

**Figure 2:**
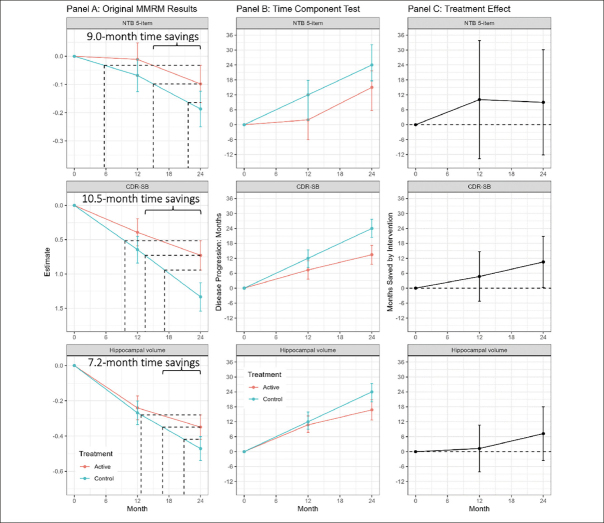
Mixed model results, time component tests, and time savings with intervention in the LipiDiDiet 2-year dataset Footnote: Panel A: Least square means (+/− SE) over time for the NTB 5-item, CDR-SB, and MRI hippocampal volume based on the pre-specified sensitivity MMRM analysis in the LipiDiDiet 2-year dataset. Panel B: Months (mean + /− SE) of disease progression relative to the control group disease progression over time. Panel C: Months (mean and 95% confidence interval) saved by intervention compared to the control group over time based on the TCT analyses in Panel B. Abbreviations: SE: standard error; NTB: neuropsychological test battery; CDR-SB: clinical dementia rating – sum of boxes; MRI: magnetic resonance imaging; MMRM: mixed model for repeated measures.

LipiDiDiet was a randomized, controlled, doubleblind, parallel-group, multicenter trial designed to assess the safety and efficacy of Fortasyn Connect (Souvenaid), a once-daily nutritional supplement drink, in individuals with prodromal AD according to IWG1 criteria (15, 16). The primary outcome of the study was a 5-item composite Neuropsychological Test Battery (NTB) z-score which is a cognitive measure. Secondary outcomes included the Clinical Dementia Rating Sum of Boxes (CDR-SB) (a global assessment of cognition and function), and a structural atrophy outcome of total hippocampal volume from magnetic resonance imaging (MRI). The primary timepoint for the study was 24 months. In the pre-specified sensitivity MMRM analysis of the LipiDiDiet study, which does not assume linearity of the treatment effect, the mean difference between Fortasyn Connect and placebo in the change from baseline to endpoint (24 months) on the 5-item composite NTB was 0.089 (95% confidence interval (CI) −0.090, 0.268; Fig 2, top left). An important secondary outcome of LipiDiDiet was the CDR-SB which had a difference of −0.605 in the change from baseline at 24 months (95% CI −1.184, −0.026; Fig 2, middle left). MRI hippocampal volume had a point difference of 0.122 at 24 months (95% CI −0.067, 0.311; Fig 2, lower left).

Using the LipiDiDiet dataset, the TCT was applied to these 3 outcomes – the 5-item NTB, the CDR-SB and hippocampal volume – individually, and also with all 3 outcomes combined in an optimized gTCT.

## Results

**Figure 3:**
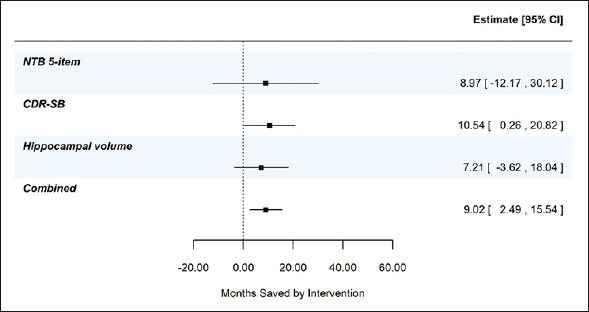
Combined evidence from gTCT in addition to summary findings from meta time component tests (TCTs) applied to the NTB 5-item, CDR-SB, and hippocampal volume; data from the LipiDiDiet 2-year dataset Based on 24-month datapoint in the LipiDiDiet 2-year dataset. Abbreviations: SE: standard error; NTB: neuropsychological test battery; CDR-SB: clinical dementia rating – sum of boxes; MRI: magnetic resonance imaging; MMRM: mixed model for repeated measures.

Figure 2, panel A shows the least squares means (+/− standard error) by treatment over time for the 5-item NTB, the CDR-SB, and MRI hippocampal volume, based on pre-specified MMRM analyses. At 24 months, disease progression was delayed by 9.0 months (95% CI −12.2, 30.1 months), 10.5 months (95% CI 0.3, 20.8 months), and 7.2 months (95% CI −3.6, 18.0 months) as measured by the 5-item NTB, CDR-SB, and hippocampal volume respectively. Figure 2, panel B shows time component translations relative to placebo progression and panel C shows time saved with Fortasyn Connect treatment relative to placebo at various time points during the study. Based on results from the MMRM analyses of the three endpoints combined in a gTCT, disease progression was delayed by 9.0 months (95% CI 2.5, 15.5 months) with 24 months of treatment (Supplemental Figure 1). The baseline to endpoint (24-month) findings for all 3 outcomes individually and the global statistical test that combines the 3 outcomes are summarized in Figure 3.

## Discussion

Assessing the clinical meaningfulness of early interventions in AD is a multifaceted and complex issue that must be considered based on the idiosyncratic aspects of the trial. Simply put, there is no universally acceptable measure to express the magnitude of treatment effect on any outcome to assess if it is meaningful or not. Therefore, a metric that is easily understood by diverse stakeholders is useful so that the determination of whether a treatment effect is meaningful or not to a particular stakeholder and can be widely applied across the varying scenarios.

Studies in AD have traditionally focused on the difference between treatments in mean change from baseline to endpoint on a validated scale. However, expressing results via this summary measure are open to misinterpretation, because it is hard to contextualize a mean difference when mean changes in the control arm are small. Mean point differences are more easily interpreted when summarized as percent slowing, when this is a relative measure of the vertical distance between treatments. For LipiDiDiet the benefits observed equated to 0.089 with Fortasyn Connect for the 5-item NTB primary outcome, −0.605 for the CDR-SB outcome and 0.122 cm3 for MRI hippocampal volume atrophy. However, using time saved as the summary measure, as done in TCTs, provides an absolute measure of the horizontal distance between treatments and yields a practical interpretation of the treatment effect that is relevant without requiring any further information about the scale.

Expressing the study finding as a percent slowing can address some of the misunderstanding around difference in absolute point change between treatment groups (i.e., point change with active treatment as a percent of that seen with placebo) (4), but lack of clear, intuitive interpretation for ‘slowing’ of disease progression still allows potential for confusion. If the decline over time in the placebo group is linear, then percent slowing of the points on the clinical scale will equate to time savings, however, with non-linearity, a direct calculation of time saved is critical to an appropriate interpretation of the results.

Converting to the well-understood metric of time and expressing treatment effect as time saved can further facilitate the broader understanding of clinical trial findings. This time metric is closely associated with what the patient and their care partner want to know, i.e., how long will treatment maintain the patient’s current functioning and delay transition to a more advanced stage of disease with less independence and increased burden on the care partner? The TCT additionally has the benefit of facilitating comparisons across studies that employed different outcome measures with the caveat that other trial differences must be considered (e.g., patient population, time of treatment). It is important to note that Alzheimer’s disease symptoms are diverse, and progression of some of these symptoms is not uniformly worsening. For example, specific segments of progression can be associated with transient depression or agitation. This suggests that the value of progression slowing therapies may be greatest in the earliest stages of disease.

In this study, we employed the LipiDiDiet RCT on the effects of the multi-nutrient combination Fortasyn Connect and updated it with meta time TCT to investigate the meaningfulness of clinical benefits. Our findings demonstrate that the metric of ‘time saved’ offers valuable insight into this complex matter. The results indicate that relying solely on absolute or relative values that measure the vertical distance between treatments, such as a 45% disease slowing on the CDR-SB scale or a 0.122 point reduction in hippocampal atrophy relative to placebo, is less informative than measuring the horizontal distance in ‘time saved,’ such as 10.5 months saved out of 24 months for CDR-SB – corresponding to a 44% slowing of disease time – or 9.0 months saved out of 24 months for cognitive performance as in the 5-item NTB – corresponding to a 38% slowing of disease time. The ‘time saved’ appears more accessible and meaningful for individual patient care decisions.

The TCT is derived from mean changes on clinical scales and as such the assumptions inherent to the mean change analysis and the attributes of the clinical scale also apply to the TCT. The TCT is not a new outcome but rather a new approach to expressing existing outcomes in a meaningful way. As such, outcomes that are highly variable on the actual scale will also be highly variable on the time scale. The TCT cannot contrive a greater level of certainty or a greater magnitude of treatment effect through some analytic trick or assumption. Therefore, if for example, the field considers an MMRM analysis of the CDR-SB as an acceptable analysis and outcome measure for a certain scenario, a TCT derived from an MMRM analysis of the CDR-SB would be acceptable too. While simulation studies suggest that TCTs have well-controlled type I error and comparable power to the original scale (21), inferences on the original scale and time scale would not be expected to agree exactly. The choice of which analysis will serve as the primary for interpretation of statistical significance should be decided in advance. Prospective validation of the proposed TCT approaches will be essential.

An area of particular focus in AD trials is the shape (functional form) of the mean changes over time. An analysis that does not assume linear trends over time is often preferred because the assumption of linearity can be difficult to justify a priori (23). Therefore, in the present analysis for LipiDiDiet the prespecified sensitivity analysis in which no assumption on linearity is made was used here as the basis for deriving the TCT. This analysis suggested that mean changes did not follow a linear trend over time.

Another consideration in interpreting results is that the magnitude of treatment effect can vary across outcomes. If mean change results differ across outcomes, TCT results will also differ across outcomes. It can be useful to apply a global statistical testing framework in which results are combined across outcomes. It is especially straightforward to apply global tests to TCTs because the various outcomes are already on the same scale – time.

In the current study, we applied the meta TCT to the 5-item NTB, the CDR-SB and hippocampal volume. Though individual outcomes provide valuable information on the treatment effect, combining TCTs across outcomes created a more robust and accurate summary of time saved. This is shown in Supplemental Figure 1 where less variability and more precision (narrower confidence interval) was seen in the combined gTCT across the 3 outcomes – 9 months of time savings (95% CI 2.5, 15.5 months) relative to placebo over 24 months of treatment.

Here, we have adopted a simple and transparent meta TCT, based on horizontal projections onto the estimated placebo mean trajectory. The language and importance of measuring disease progression for an investigational agent in comparison to a control in terms of “time savings” has gained traction with regulators – see, for example, Leqembi advisory committee minutes (24) – due to the transparency of interpretation and inherent meaningfulness of time. Others have begun to explore this concept and its advantages. For example, Raket (2022) (5) recently estimated treatment effects as slowing of progression (i.e., time metric) using progression models for repeated measures (PMRM). These alternative methods are expected to give similar results to the presented horizontal projection meta TCTs. Additionally, construction of TCTs based on individual participant data is an area of active research.

In conclusion, it is more natural to comprehend and more clinically applicable to provide information on treatment effect as a time metric (i.e., months saved). Understanding whether a treatment effect is clinically meaningful in terms of a time metric rather than difference in absolute or percent point change on a scale provides more clarity to the clinician, patient, care partner, and to payers. Additionally, converting to a time component facilitates easier comparison of findings across studies, subject to other study differences.

## Notes

*Ethical standards:* The study was approved by ethics committees of all sites and done in accordance with the Declaration of Helsinki and International Conference on Harmonization Good Clinical Practice guidelines.
